# Acute Normobaric Hypoxia Lowers Executive Functions among Young Men despite Increase of BDNF Concentration

**DOI:** 10.3390/ijerph191710802

**Published:** 2022-08-30

**Authors:** Maciej Chroboczek, Sylwester Kujach, Marcin Łuszczyk, Tomasz Grzywacz, Hideaki Soya, Radosław Laskowski

**Affiliations:** 1 Department of Physiology, Gdansk University of Physical Education and Sport, 80-336 Gdansk, Poland; 2 Department of Sport, Institute of Physical Education, Kazimierz Wielki University, 85-064 Bydgoszcz, Poland; 3 Sports Neuroscience Division, Advanced Research Initiative for Human High Performance, Faculty of Health and Sport Sciences, University of Tsukuba, Tsukuba 305-8574, Japan; 4 Laboratory of Exercise Biochemistry and Neuroendocrinology, Department of Sports Neuroscience, Advanced Research Initiative for Human High Performance (ARIHHP), Faculty of Health and Sports Sciences, University of Tsukuba, Ibaraki 305-8577, Japan

**Keywords:** cognitive function, physical exercise, altitude, cortisol

## Abstract

Background: Decreased SpO_2_ during hypoxia can cause cognitive function impairment, and the effects of acute hypoxia on high-order brain functions such as executive processing remain unclear. This study’s goal was to examine the impact of an acute normobaric hypoxia breathing session on executive function and biological markers. Methods: Thirty-two healthy subjects participated in a blind study performing two sessions of single 30 min breathing bouts under two conditions (normoxia (NOR) and normobaric hypoxia (NH), FIO_2_ = 0.135). The Stroop test was applied to assess cognitive function. Results: No significant difference was observed in the Stroop interference in the “reading” part of the test in either condition; however, there was a significant increase in the “naming” part under NH conditions (*p* = 0.003), which corresponded to a significant decrease in SpO_2_ (*p* < 0.001). There was a significant increase (*p* < 0.013) in the brain-derived neurotrophic factor (BDNF) level after NH conditions compared to the baseline, which was not seen in NOR. In addition, a significant drop (*p* < 0.001) in cortisol levels in the NOR group and a slight elevation in the NH group was noticed. Conclusions: According to these findings, acute hypoxia delayed cognitive processing for motor execution and reduced the neural activity in motor executive and inhibitory processing. We also noted that this negative effect was associated with decreased SpO_2_ irrespective of a rise in BDNF.

## 1. Introduction

Hypoxia is often linked with cognitive decline. Hypoxia SpO_2_ levels decrease when ascending to high altitudes, which, in turn, can cause cognitive impairment. However, it is still unknown how acute hypoxia affects higher-order brain functions including executive processing and how it affects the central nervous system (CNS) [1,2]. Hypoxia can cause perturbations in the CNS due to alterations in hormonal/humoral factor levels as well as neurotransmitters [3].

On the other hand, humans have at least several mechanisms responsible for improving cognitive function. Brain imaging studies have shown that one of these mechanisms that can improve cognitive performance is increased cerebral blood flow (CBF). Increased CBF (e.g., in the middle cerebral artery) has the potential to affect brain tissue and its metabolism due to enhancing the brain’s supply of glucose, oxygen, and peripherally produced hormones [4].

Many of these hormones act as a neurochemical and play a sufficient role in brain plasticity. One of the more crucial ones is brain-derived neurotrophic factor (BDNF). BDNF induces a cascade of events via receptor kinase B (TrkB), which can promote the functional and structural plasticity of the brain [5]. It is currently considered one of key proteins that can modulate cognitive functions [6]. It is also crucial for memory consolidation and for creating additional neural connections, etc. [7,8,9]. This may be explained by the ability of BDNF to cross the blood–brain barrier bidirectionally according to a concentration gradient [10]. Additionally, BDNF can be a biomarker for impaired memory in humans, as shown in a recent study [11]. On the other hand, it can cause cognitive enhancements, which are connected to higher BDNF expression in the hippocampus, dentate gyrus, and perirhinal cortex [12]. The data show that, when BDNF expression in the brain is increased, its blood level can also be observed [13,14]. The mentioned increase of the cerebral origin neurotrophic factor level influences brain neurogenesis. Improvement in cognitive functions has also been detailed [15,16,17]. In turn, decreased levels of neurotrophin may lead to an impaired ability to remember assigned tasks, mainly through exposure to stress or depressive conditions [18].

The hypothalamic–pituitary–adrenal axis is responsible for a whole symphony of actions when the body encounters so-called stressors and environmental differences, and can be counted as one. They can significantly affect cognitive functions due to the stress reaction, and determining the level of cortisol could help estimate the stress level [19,20]. Cortisol is one of the main glucocorticoids, produced in the adrenal gland cortex, and has a wide metabolic effect [21,22,23]. Some studies show cortisol rises after hypoxic exposure [24,25], although on the other hand, there are some studies showing no effect after such exposure [26,27].

The purpose of this study was to answer the question of whether acute normobaric hypoxia exposure will affect executive functions, peripheral BDNF concentrations, and stress level through cortisol. It was hypothesized that executive functions will improve after intervention and that this improvement will appear due to rise of BDNF concentration independent of changes in the cortisol level. It was examined how executive function was affected after acute exposure to hypoxic gas mixtures with FIO_2_ = 13% oxygen concentration.

## 2. Materials and Methods

Information about the experiment was provided by members of the research team through posters and short presentations. The study involved Gdansk University of Physical Education and Sport students (*n* = 32, all men, aged 20.4 ± 0.6). The subjects participated in a single blind study where they performed two separate sessions of 30 min breathing bouts, under two conditions (normoxia (NOR) and normobaric hypoxia (NH)) on different days. One week prior to the intervention, the participants underwent familiarization with all laboratory equipment and performed trial tests. On the next visit, the experiment began. The first session consisted of ambient air breathing. On the second session, the participants breathed hypoxic air (fraction of inspired oxygen (FIO_2_) = 0.135), which corresponded to an altitude of 3500 m. The breathing sessions were separated by a two-week break. Before and after both sessions, the participants performed a color-word Stroop task (which lasted an average of 5–6 min) and immediately afterwards were subjected to a blood draw. Two participants were tested at one time, with a staggered time allowing for cognitive testing and blood draws to avoid queuing and to standardize the testing conditions. During inhalation, one research team member per participant observed their condition and the level of SpO_2_. To assess BDNF serum concentrations and estimate cortisol levels, the ELISA method was applied (a description is provided in the ‘Blood Sampling’ section). Cognitive testing included a Stroop interference test (cognitive control). Written consent was obtained from all participants before executing the experimental protocol. The participants were verbally informed of the possibility of opting out of the study. The research was approved by the Local Ethics Committee and the Bioethical Committee of the Regional Medical Society (KB-9/16) according to the Helsinki Declaration. The participants did not have any medical contraindications. Detailed participant characteristics are shown in Table 1.

### 2.1. Anthropometric Measurements

To measure body height, an anthropometer from a GPM measuring set was used (Skinfold Caliper User’s Manual—Poland). A TBF-300 Tanita Body Fat Monitor/Scale Analyzer (Japan) was used to assess body mass and body composition (body fat (FAT); fat-free mass (FFM)). To evaluate overall body build, body mass index (BMI) (kg∙m^−2^) was used.

### 2.2. Normobaric Hypoxia

The hypoxic gas mixture for the trials was produced by the GO2Altitude ERA II Hypoxic/Hyperoxic Air Generator from Biomedtech (Australia). Height above sea level (a.s.l.) was simulated by reducing the oxygen content of the inspiratory gas mixture according to the manufacturer’s recommendations (Biomedtech Australia Pty Ltd., Biomedical Research and Development, Belmore, Australia) and described in GO2Altitude ERA II Hypoxicator System Operational Manual [28]. The FIO_2_ = 13% oxygen level in the mixture was used to create a hypoxic mixture that replicated an altitude of 3500 m a.s.l. While breathing, the participants were unaware of the gas mixture. When conducting testing under normoxia conditions, they also had on pulse oximeters and used masks; however, at that time, the air generator only produced a sea-level breathing mixture.

### 2.3. Cognitive Functions

#### Executive Functions: Stroop Test

To measure cognitive control, the abbreviated version of the Stroop interference test from the Vienna Test System database was used. The first part involves giving “names” of colors. Part two is about “reading” color names. The third part requires giving the name of the font color with which each word was written instead of reading the written word. For example, the “blue” stimulus should be reacted with the word “red”, suppressing the natural tendency to read “blue”. Such a task requires constant control and suppression of a natural automatic response in favor of a task consciously managed and subordinated to the rules. The result usually contains several elements, including the time of each test, the difference between the time of the first and third test, and the number of errors in the third test [29]. The cognitive test was the same as in a previously published study [30].

### 2.4. Blood Sampling

Before and after the intervention, blood samples from the antecubital vein into vacutainer tubes were taken to measure the serum concentrations of BDNF and cortisol. At 4 °C and 2000× *g*, the samples were centrifuged for 10 min. Following separation, the serum samples were frozen and stored at 70 °C for further analysis. Since the most popular technique for evaluating how human growth factors in blood relate to individual variations in neuropsychiatric, cognitive, and exercise aspects, serum analysis was conducted [31]. The sample was diluted 1:5 before use. The manufacturer reported 4–6% for the intra-assay coefficients of variability (CVs) and 8–10% for the inter-assay CVs, respectively. Both BDNF and cortisol were determined via an enzyme immunoassay method using commercial kits according to the manufacturer’s instructions (R&D Systems, Minneapolis, MN, USA, catalog no. DBD00; Ray Biotech Inc., Cambridge, UK; Demeditec Diagnostics GmbH, Kiel, Germany, catalog no. DEH3388, respectively). Based on our prior experiences and recommendations from the literature, a 1 h clotting period was permitted for the proper serum BDNF dosage [32,33].

### 2.5. Statistical Analysis

The initial storage of the results and their statistical analysis were done using Microsoft Excel version 10.0 for Windows. Utilizing GraphPad Prism 7’s tools, a statistical analysis was conducted. Calculated were arithmetic means, standard deviation, and levels of significance for variances between means. The distribution of each variable was then examined using descriptive statistical techniques, including a parametric paired Student’s *t*-test. To further examine the significance of variations across groups and over time, a two-way analysis of variance (ANOVA) with repeated measurements was performed. The Bonferroni post hoc test was used to further examine significant effects. The significance for all analyses was assumed at *p* < 0.05.

## 3. Results

### 3.1. Blood Saturation under Normoxia and Acute Normobaric Hypoxia Conditions

**Figure 1:**
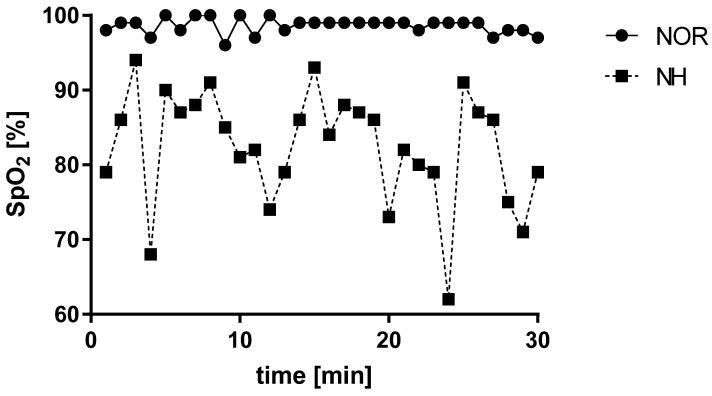
Effect of a single 30 min exposure under normoxia and normobaric hypoxia at FIO_2_ = 13% conditions on peripheral blood saturation. NOR—normoxia; NH—normobaric hypoxia. Data expressed as mean of the entire group at particular time points.

Acute exposure to normobaric hypoxia conditions revealed a decrease in blood saturation (t = 12, df = 29; *p* < 0.001). These changes were appropriate to the simulated altitude above sea level (Figure 1).

### 3.2. Stroop Test under Conditions of Normoxia and Acute Normobaric Hypoxia

**Figure 2:**
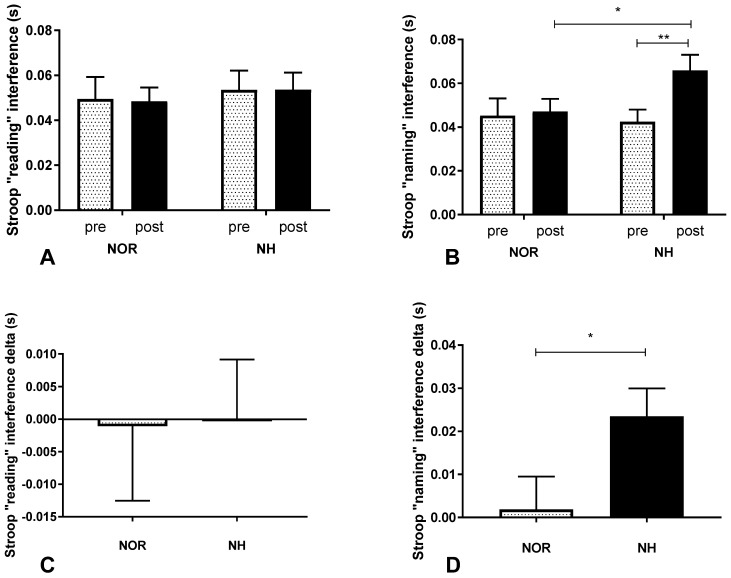
Effect of a single 30 min exposure under normoxia and normobaric hypoxia at FIO_2_ = 13% conditions on post-exposure interference values in “reading” (**A**) and in “naming” (**B**) and their deltas (**C**,**D**). Values are means. Error bars indicate SEM (standard error of the mean). * *p* < 0.05; ** *p* < 0.01.

The participants performed cognitive tests after exposure to normobaric hypoxic conditions simulating 3500 m a.s.l. There were no statistical differences in “reading” interference (interaction F (1, 60) = 0.006, *p* = 0.939; time F (1, 60) = 0.0047, *p* = 0.946) (Figure 2A). On the contrary, “naming” interference was significantly changed (interaction F (1, 60) = 4.644, *p* = 0.035; time F (1, 60) = 6.375, *p* = 0.014) (Figure 2B), which corresponded with a significant decrease in the peripheral level of SpO_2_. Next, contrast analysis between NOR versus NH (post vs. pre) was performed (Figure 2C,D).

### 3.3. Blood Analysis

**Figure 3:**
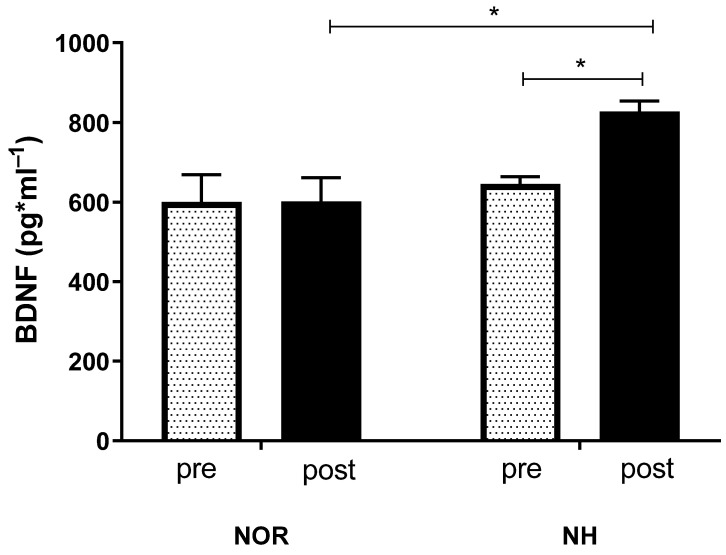
Effect of a single 30 min exposure under normoxia and normobaric hypoxia at FIO_2_ = 13% conditions on post-exposure serum BDNF concentration. Values represent means. Error bars indicate SEM. * *p* < 0.05.

The mean values of BDNF concentration did not change in the NOR conditions in pre-post measurements, but a significant rise in the concentration of BDNF after the simulated NH conditions was observed (interaction F (1, 62) = 3.893, *p* = 0.053; row factor F (1, 62) = 7.358, *p* = 0.009; time F (1, 62) = 3.999, *p* = 0.0499) (Figure 3).

**Figure 4:**
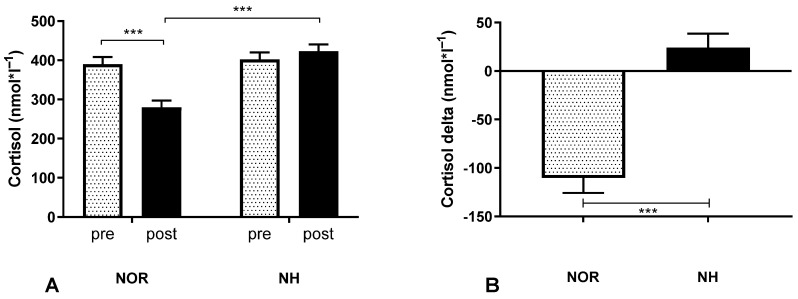
Effect of a single 30 min exposure under normoxia and normobaric hypoxia at FIO_2_ = 13% conditions on post-exposure serum cortisol concentration (**A**) and its delta (**B**). Values represent means. Error bars indicate SEM (standard error of the mean). *** *p* < 0.001.

The test analysis revealed significant changes in the cortisol values under NOR conditions in the pre-post measurements, although the same effect after simulated NH conditions was not observed. Interactions between groups occurred (interaction F (1, 62) = 35.9, *p* < 0.001; row factor F (1, 62) = 11.71, *p* = 0.001; time F (1, 62) = 16.61, *p* = 0.001), and the delta was also shown (Figure 4). Mixed model ANOVA showed the same results and they are available in the supplementary materials.

## 4. Discussion

The main goal of the present study was to examine the impact of a single session of simulated acute normobaric hypoxia on the BDNF concentration and relate to it executive functions, as well as the cortisol level in young adults.

Significant cognitive decline after a single session of acute NH breathing was noticed, which is in line with previous research [34,35,36]. This deterioration was reflected by increased interference in the Stroop test.

The timepoint of cognitive performance, which in our study occurred immediately after hypoxia exposure, is not without relevance. The reperfusion effect, which has been described in both animal models [37] and in humans [38,39,40], could improve cognitive functions at that time. This post-exposure improvement is most likely due to this reperfusion and, thus, better tissue oxygenation [41,42]. Furthermore, hypoxic-induced oxidative stress may alter BDNF production and/or its release, preventing hippocampal impairment [43,44,45]. As a neuro-regulator and factor influencing brain neurogenesis, an increase in the level of the cerebral neurotrophic factor and, consequently, an improvement in cognitive functioning has also been reported [15,16,17].

Although an increase in BDNF concentration was observed, a decline in cognitive function after 30 min of acute exposure to normobaric hypoxia at a simulated altitude of 3500 m (FIO_2_ = 13%) was also noticed. This decrease is consistent with earlier research [36,46]. Cerebral hypoxia, which can be caused by arterial oxygen deficiency and decreased cerebral blood flow, is thought to play a role in executive function impairment. This impairment is related to the level of SpO_2_, which drops proportionally due to the severity of the hypoxic conditions, as we have shown in a previous study [30]. In addition, since it is strongly connected with prefrontal oxygenation, it can have an impact [47,48]. This is consistent with earlier research showing that cognitive function declines as the severity of hypoxic conditions increases [34], and that SpO_2_ can alter prefrontal oxygenation and result in CBF restriction through cerebral vasoconstriction [49].

Furthermore, our findings seem to confirm earlier studies that hypoxia had little or no impact on the post-exposure increase in CBF, which could counteract or minimize the adverse effects of hypoxia [50]. This is only speculation, since we did not measure it directly. This effect is seen in the results of the cognitive tests used in our study. Reading is thought to be a more instinctive response than naming because it is less controlled. When circumstances require that the controlled naming response prevails over the automatic reading response, it slows down [51]. Inhibition is thought to prevent participants in the Stroop paradigm from directing their attention to the irrelevant stimulus component, allowing them to concentrate on the important dimension (i.e., the color of ink in which the word is written, rather than the name of the word) [51]. Therefore, a decrease in inhibitory control would result in greater Stroop interference. Both behavioral and electrophysiological data seem to support such an interpretation [52].

Decreases in cognitive function induced by environmental changes noted as a stressor may also be reflected in biological markers. Hypoxia can activate the gland cortex to release cortisol, which has been identified as a presumed endocrinological mediator that can affect brain function [21]. Nonetheless, the results of hypoxia investigations are inconsistent in terms of the observed reactions. The most common response to these environmental stimuli is a rise in cortisol levels [53], although no changes have been documented [54]. This difference could be due to the different research designs (i.e., normobaric vs. hypobaric hypoxia). The participants’ cortisol levels were considerably lower following exposure to NOR than under NH conditions in our study. Due to the lack of difference at baseline and the non-significant increase in concentrations after exposure to NH conditions, we can speculate that the reduction in the NOR group may have been due to calming down/entering a state of relaxation by breathing calmly. On the other hand, as this was still at the start of the study, perhaps a defense mechanism to an impending unknown stimulus triggered significant changes at the biochemical level. This seems to effectively counteract the sudden cortisol level increase in the NH group, which is a possibility, considering that a stress trigger could result in such a response, especially when this hormone has the potential to impair the prefrontal cortex [22]. Some may argue that because this area of the brain regulates so many of our cognitive activities, the negative influence of cortisol on psychomotor performance is negligible, yet it seems to be one of the elements that can be thoroughly investigated in future research.

A few limitations of this study must be mentioned. Future research groups could be enlarged to assess, for example, age or sex differences. To make the experimental conditions less predictable, the sequence of NOR and NH could be more randomized. Alternately, a control group that received the NH intervention twice in a row might be used. Further studies should include brain tissue oxygenation measurement to investigate the potential mechanism responsible for these changes. This study revealed that a single session of simulated acute normobaric hypoxia leads to a significant impact on BDNF concentrations as well as cognitive impairment in the “naming” aspect of the Stroop test. Moreover, this attenuation can be likely linked to cortisol levels, although this requires further research.

## 5. Conclusions

The consequences of acute hypoxia on cognitive function are still debatable. However, hypoxia has the potential to impair cognition. The effects of a single acute exposure to simulated moderate normobaric hypoxia conditions were investigated and impairments in executive functions were observed. A reduced SpO_2_ level was associated with this detrimental effect irrespective of the rise in BDNF. Although higher BDNF levels can induce positive changes through improvements in human cognition, further research about acute hypoxia, exercise/training in such conditions, and its effect on cognition need to be conducted. According to these findings, acute hypoxia delayed cognitive processing for motor execution and reduced the neural activity in motor executive and inhibitory processing.

## Figures and Tables

**Table 1:** Anthropometric characteristics of the participants.

N = 32	X	SD
Age (years)	20.4	0.6
Height (cm)	178	7.1
Weight (kg)	76.6	11.4
FAT (%)	18.6	4.1
FAT (kg)	14.4	5.0
FFM (kg)	62.1	7.9
BMI (kg∙m^−2^)	23.9	2.4

X—mean average; SD—standard deviation; FAT—adipose tissue; FFM—free fat mass; BMI—body mass index.

## Data Availability

The data presented in this study are available on reasonable request from the corresponding author.

## Supplementary Materials

The following are available online at https://www.mdpi.com/article/10.3390/ijerph191710802/s1, Table S1: Results of the mixed-model ANOVA.
