# Supplementary material for: Acute Normobaric Hypoxia Lowers Executive Functions among Young Men despite Increase of BDNF Concentration
Source: Int J Environ Res Public Health. 2022 Aug 30;19(17):10802. doi: 10.3390/ijerph191710802 (PMC9518314; doi:10.3390/ijerph191710802)
Supplement: Supplementary file 1 [file ijerph-19-10802-s001.zip › ijerph-1857949-supplementary.pdf]

Table S1. Results of the Mixed-Model ANOVA.

	**Fixed Effects (Type III)**	**F**
**BDNF**		
Row Factor	0.0086	7.358
Time	0.0499	3.999
Row Factor x Time	0.0529	3.893
Chi-square, df	0.4329, 1	
**Cortisol**		
Row Factor	0.0011	11.71
Time	0.0001	16.61
Row Factor x Time	<0.0001	35.90
Chi-square, df	30.42, 1	
**Stroop "reading" interference**		
Row Factor	0.6097	0.2634
Time	0.9457	0.004675
Row Factor x Time	0.9387	0.005959
Chi-square, df	2.282, 1	
**Stroop "naming" interference**		
Row Factor	0.3213	1.000
Time	0.0142	6.375
Row Factor x Time	0.0352	4.644
Chi-square, df	12.53, 1	
